# Upregulation of the Transient Receptor Potential Ankyrin 1 Ion Channel in the Inflamed Human and Mouse Colon and Its Protective Roles

**DOI:** 10.1371/journal.pone.0108164

**Published:** 2014-09-29

**Authors:** József Kun, István Szitter, Ágnes Kemény, Anikó Perkecz, László Kereskai, Krisztina Pohóczky, Áron Vincze, Szilárd Gódi, Imre Szabó, János Szolcsányi, Erika Pintér, Zsuzsanna Helyes

**Affiliations:** 1 Department of Pharmacology and Pharmacotherapy, Medical School, University of Pécs, Pécs, Hungary; 2 Molecular Pharmacology Research Group, János Szentágothai Research Center, University of Pécs, Pécs, Hungary; 3 Department of Pathology, Medical School, University of Pécs, Pécs, Hungary; 4 1st Department of Internal Medicine, University of Pécs, Pécs, Hungary; St. Joseph's Hospital and Medical Center, United States of America

## Abstract

Transient Receptor Potential Ankyrin 1 (TRPA1) channels are localized on sensory nerves and several non-neural cells, but data on their functional significance are contradictory. We analysed the presence and alterations of TRPA1 in comparison with TRP Vanilloid 1 (TRPV1) at mRNA and protein levels in human and mouse intact and inflamed colons. The role of TRPA1 in a colitis model was investigated using gene-deficient mice. TRPA1 and TRPV1 expressions were investigated in human colon biopsies of healthy subjects and patients with inflammatory bowel diseases (IBD: ulcerative colitis, Crohn's disease) with quantitative PCR and immunohistochemistry. Mouse colitis was induced by oral 2% dextran-sulphate (DSS) for 10 days. For investigating the functions of TRPA1, Disease Activity Index (weight loss, stool consistency, blood content) was determined in C57BL/6-based Trpa1-deficient (knockout: KO) and wildtype (WT) mice. Sensory neuropeptides, their receptors, and inflammatory cytokines/chemokines were determined with qPCR or Luminex. In human and mouse colons TRPA1 and TRPV1 are located on epithelial cells, macrophages, enteric ganglia. Significant upregulation of TRPA1 mRNA was detected in inflamed samples. In Trpa1 KO mice, Disease Activity Index was significantly higher compared to WTs. It could be explained by the greater levels of substance P, neurokinins A and B, neurokinin 1 receptor, pituitary adenylate-cyclase activating polypeptide, vasoactive intestinal polypeptide, and also interleukin-1beta, macrophage chemoattractant protein-1, monokine induced by gamma interferon-1, tumor necrosis factor-alpha and B-lymphocyte chemoattractant in the distal colon. TRPA1 is upregulated in colitis and its activation exerts protective roles by decreasing the expressions of several proinflammatory neuropeptides, cytokines and chemokines.

## Introduction

Inflammatory bowel diseases (IBD), including ulcerative colitis (UC) and Crohn's disease (CD), are the most common chronic inflammatory disorders of the intestine. UC affects only the colon, CD may affect all parts of the gastrointestinal tract, but most commonly the distal part of the small intestine, the ileum, and the colon. Clinical symptoms of IBD comprise of abdominal pain, diarrhea, gastrointestinal bleeding and weight loss [1]. Dextran sodium sulphate (DSS)-induced mouse colitis is one of the most widely used, non-genetic models of IBD. Through chemically damaging the epithelial barrier, DSS induces colonic inflammation and ulceration leading to progressive crypt loss in the colonic mucosa, alterations of luminal bacterium species and activation of inflammatory cells [2], [3].

IBD can be a painful and debilitating disease of the digestive tract with potentially life-threatening complications. Available immunosuppressive therapies are associated with potential significant adverse effects and there remains a cohort of patients with refractory or relapsing disease. This poses a driving force to understand the complex pathophysiological mechanisms, identify key mediators and find novel therapeutic targets [4].

Capsaicin, the pungent principle of red pepper, selectively excites a subpopulation of afferents called capsaicin-sensitive sensory neurons [5], [6] that densely innervate the gastrointestinal (GI) tract [7], [8]. Peptide transmitters, e.g., substance P (SP), neurokinin A (NKA), calcitonin gene-related peptide (CGRP) [9] are released from these fibres upon their activation. The specific receptor for capsaicin is the Transient Receptor Potential Vanilloid 1 (TRPV1) [6], [10]. TRPV1 expressing extrinsic sensory neurons are involved in intestinal inflammation, however, the role of TRPV1 remains controversial in the pathogenesis of IBD [11]–[18]. Transient Receptor Potential Ankyrin 1 (TRPA1) is a similar receptor-ion channel complex in terms of structure, function and localization [19]. It has been well established that TRPA1 and TRPV1 are extensively coexpressed in a subpopulation of peptidergic, afferent Aδ- and C-fibers whose cell bodies lie in dorsal root, trigeminal, and nodose/jugular ganglia [20]–[23]. Thirty to fifty percent of TRPV1-expressing neurons contain TRPA1 while the latter rarely exists in neurons without TRPV1 [24], [25]. In the brain, TRPV1 has been widely detected [26]–[28] and data are accumulating for the presence of TRPA1, as well [28]–[30]. TRPA1 is generally colocalized with TRPV1 also in non-neuronal cells (e.g., epithelial cells) [31]. TRPA1 functions as a Ca^2+^-permeable non-selective cation channel in different cellular processes from sensory to homeostatic functions. TRPA1 is activated by noxious cold (<17°C), mechanical stimuli and various electrophilic, irritant, and pungent compounds, some of them could be found in the human diet (e.g., allylisothiocyanate, allicin, cinnamaldehyde, menthol). TRPA1 is also activated/sensitized by mediators of inflammation, oxidative stress and tissue damage [32]–[35]. In the GI tract, TRPA1 occurs in distinct systems: extrinsic primary afferent neurons, intrinsic enteric neurons, endocrine cells of the mucosa and mucosal epithelial cells [7], [8], [19]. TRPA1 acts as a chemosensor in the gut detecting the luminal environment and modulating gastrointestinal functions, such as nociception, gastric tone, spicy diet-induced satiety, GI motility, and secretory effects [19].

Upon TRPA1 activation, inflammatory tachykinins (substance P: SP and neurokinin A: NKA), and calcitonin gene related peptide (CGRP) are released from sensory nerve endings [36] and induce neurogenic inflammation characterized by vasodilatation, plasma extravasation, edema, leukocyte activation and migration [6], [37], [38]. Intracolonic administration of trinitrobenzenesulfonic acid (TNBS) or DSS in mice leads to severe colitis with a neurogenic component: calcitonin gene related peptide (CGRP) and SP are released from extrinsic sensory neurons [39]–[41]. The role of CGRP has been shown to be protective in colitis [39], [40]. There are data for the colitis-inducing and maintaining roles of SP in the DSS colitis model, as well as UC patients [39], [42], [43]. The NK1 receptor of substance P was found to be upregulated in the colon after induction of colitis in rodents and in human IBD [41], [44], [45]. We found that both pharmacological blockade and genetic deletion of the NK1 receptor attenuated DSS colitis in mice [44].

Although TRPA1 activation induces SP-release, a complex role is suggested for the receptor in IBD [46]. Data on the function of TRPA1 activation in experimental colitis are in fact contradictory, it has been reported to be proinflammatory, antiinflammatory or without any effect [39], [47]–[49]. Activation of sensory TRPA1 can lead to the release of antiinflammatory peptides, such as somatostatin, eliciting a systemic counterregulatory “sensocrine” function [50], [51]. Complicating the picture, the role of non-neuronal TRPA1 receptors is not understood yet [31].

Therefore, the objectives of the present study were *1.* to investigate the local, including non-neuronal mRNA expression level and protein localization of TRPA1 in comparison with the TRPV1 in the intact and inflamed mouse distal colon and colon biopsy samples of IBD patients; *2.* to study the role of TRPA1 in DSS-colitis using gene-deleted mice and analyzing the TRPA1-mediated changes in cytokine, neuropeptide and neuropeptide receptor expressions. Comparing the significant pathological signs of DSS-induced murine colitis with the human data we aimed to shed light on the translational relevance of our findings.

## Materials and Methods

### Animals

The 10-day DSS treatment was performed on male TRPA1 gene-deficient (knock-out, KO) mice [52] backcrossed for 8-10 generations to C57BL/6 mice, and their wildtype (WT) counterparts. The original heterozygous breeding pairs (Trpa1^+/−^) were kindly provided by Pierangelo Geppetti (University of Florence). They were then bred on as separate Trpa1^+/+^ and Trpa1^−/−^ lines in the Laboratory Animal House of the Department of Pharmacology and Pharmacotherapy of the University of Pécs at 24–25 °C, provided with standard mouse chow and water ad libitum and maintained under a 12-h light-dark cycle. Mice were 8-10 weeks old and weighed 22–25 g. The first three generations of offsprings of the donated *Trpa1* ^+/−^ mice and also the animals used in the experiment were genotyped by PCR analysis [53]. For the completed *Animal Research: Reporting In Vivo Experiments* (ARRIVE) Guidelines Checklist please see Table S3.

### Patients

Human colon biopsies were derived from: 1. patients with a non-inflammatory condition (diverticulosis) or healthy subjects receiving a check-up examination (n = 5 per group); 2. patients with colon tumors (polypus coli, adenocarcinoma; n = 8); 3. patients with ulcerative colitis or Crohn's disease (n = 12). Samples were stored in RNALater (Sigma-Aldrich Ltd, Budapest) in −80°C for qPCR. Samples for immunhistochemistry were fixed in formaldehyde.

### Induction of colitis

Colitis was induced with 20 mg/ml DSS (MP Biochemical) dissolved in the drinking water and administered for 10 days to WT and TRPA1 KO male mice kept in their homecages in our laboratory. WT and KO animals were randomly allocated into respective tap water-receiving control groups (1 control group per genotype, n = 5 per group). The number of DSS-receiving mice was n = 9 WT and n = 9 KO groups, 3 mice per genotype were randomly selected from the DSS-receiving animals after 3, 7 and 10 days of the treatment. A single animal served as the experimental unit. Then mice were sacrificed in deep ketamine-xylazine anaesthesia (100 mg/kg ketamine i.p.; Richter Gedeon Ltd, Budapest, Hungary, 5 mg/kg xylazine i.p.; Eurovet Animal Health BV, The Netherlands) after fasting overnight. After the DSS treatment, distal colon samples (one third of the colon from anus to cecum) were dissected for gene expression, cytokine concentration determination and histopathological evaluation.

### Disease Activity Index (DAI) assessment

The clinical symptoms of colitis, such as body weight change, stool consistency and fecal blood content, were scored on a daily basis. Fecal blood was assessed with the Hemocare test that uses a modified guaiac method (Care Diagnostica, Austria). The detailed scoring system is shown in Table S1. Scores for the 3 parameters were averaged for each mouse to obtain the Disease Activity Index [44]. For the scoring chart see Table S1 in the Supporting Information.

### Histological evaluation

The distal colon samples were fixed in 40 mg/ml buffered formaldehyde, embedded in paraffin, sectioned (5 µm), and stained with haematoxylin and eosin. Digital micrographs were taken by an Olympus BX51 microscope and Olympus DP50 camera. Inflammatory alterations were evaluated and scored by an expert pathologist blinded from the experimental design. For the histopathological semiquantitative scoring chart [44] see Table S2 in the Supporting Information.

### Immunohistochemistry

Deparaffinized and rehydrated tissue sections were incubated in acidic (pH 6) citrate buffer in a microwave oven for 3×5 min (750 W) for antigen retrieval. Endogenous peroxidase activity of the tissue was inhibited by 20 min of incubation with 3% hydrogen peroxide. Non-specific binding of the secondary antibody was blocked with normal goat serum for 20 min. Three slides per human and three slides per mouse sample were incubated with polyclonal primary antibodies for 1 hour as follows. Mouse anti-CD68 (KP1) antibody: ab125212 (Abcam, Cambridge, UK) in 1∶300 dilution. Mouse and human TRPA1 antibody: ab68847 in 1∶300 dilution, specificity tested (Figure S1) by preadsorption with immunizing peptide ab150297 in 1∶100 dilution (Abcam, Cambridge, UK). Mouse TRPV1 antibody: ab74813 in 1∶300 dilution, specificity tested (Figure S1) by preadsorption with immunizing peptide ab190844 in 1∶100 dilution (Abcam, Cambridge, UK). Human TRPV1 antibody: GP14100 in 1∶100 dilution, specificity tested (Figure S1) by preadsorption with immunizing peptide P14100 in 1∶10 dilution (Neuromics, Edina, MN, USA). Then, the slides were incubated with the respective secondary EnVision system anti-rabbit or anti-guinea pig secondary antibody conjugated with horseradish peroxidase (Dako-Cytomation, Dako North America, Carpinteria, CA, USA) for 30 min. Finally, immunolocalization of the target receptor was detected by diaminobenzidine development for 10 min, and nuclear staining was performed with hematoxylin.

### Quantitative PCR

Human biopsy samples and one third of each mouse distal colon sample were stored in RNAlater (Sigma-Aldrich Ltd., Budapest) in −80°C and homogenized in 1 ml of TRI Reagent (Molecular Research Center, Inc., Cincinnati, OH, USA). Isolation of total RNA was carried out according to the manufacturer's protocol up to the step of aquiring the aqueous phase. Briefly, distal colon tissue samples were homogenized in 1 ml of TRI Reagent, then 200 µl of bromo-chloro-propane (BCP, Molecular Research Center, Inc., Cincinnati, OH, USA) was added. RNA was purified from the aqueous phase using the Direct-zol RNA MiniPrep kit (Zymo Research, Irvine, CA, USA) according to the manufacturer's protocol. Briefly, 400 µl of the aqueous phase was mixed with 400 µl absolute ethanol, the mixture was loaded into the column, washed and the RNA was eluted in 50 µl of RNase-free water. The quantity and purity of the extracted RNA was assessed on Nanodrop ND-1000 Spectrophotometer V3.5 (NanoDrop Technologies, Inc., Wilmington, DE, USA). 1 µg of total RNA was reverse-transcribed into cDNA using the Maxima First Strand cDNA Synthesis Kit (cat. no. K1642, Thermo Fisher Scientific, Waltham, MA, USA) following the manufacturer's instructions. The obtained cDNA samples were amplified using the MX3000P qPCR system (Agilent Technologies, Santa Clara, CA, USA) and Maxima Master Mix (#K0221 for SYBR Green, #K0231 for probe detection, Thermo Scientific, Waltham, MA, USA). PCR cycle parameters were set as instructed by the manufacturer of the master mix. For assays used see Table 1. Beta-glucuronidase (GUSB) and glyceraldehyde 3-phosphate dehydrogenase (GAPDH) served as internal reference genes and the geometric mean of their threshold cycle (C_t_) values was used for normalization. The efficiency values of primers were determined to be between 95-105%. cDNA samples were measured in duplicates and the geometric means of duplicate C_t_ values were calculated to reduce technical variability and to show biological variability. Relative gene expression was calculated by the ΔΔC_t_ method.

**Table 1:** Human and mouse assays used in qPCR detection.

	Gene	Accession	Assay
**Human**	GAPDH	NM_001256799 NM_002046	(2) Hs02758991
	GUSB	NM_000181	(2) Hs00939627_m1
	Trpv1	NM_018727 NM_080704 NM_080705 NM_080706	(2) Hs00218912_m1
	Trpa1	NM_007332	(2) Hs00175798_m1
**Mouse**	Gapdh	NM_008084	(1) Mm.PT.39a.1
	Gusb	NM_010368	(1) Mm.PT.39a.22214848
	Trpv1	NM_001001445	(1) Mm.PT.56a.13426135
	Trpa1	NM_177781	(2) Mm00625268_m1
	Vip	NM_011702	(1) Mm.PT.56a.32346790
	Adcyap1 (PACAP)	NM_009625	(1) Mm.PT.56a.31241204
	Sst	NM_009215	(2) Mm00436671_m1
	Cxcl13 (BLC)	NM_018866	(1) Mm.PT.56.31389616
	Il1rn (IL-1RA)	NM_001039701	(1) Mm.PT.56.43781580
	Tac1	NM_009311	(3) F 5′- AAATGTGCGCTATGAGGAATGA -3′ R 5′- GGAAACATGCTGCTAGGAATACAAA -3′
	Tac3	NM_001199971	(3) F 5′-TCTGGAAGGATTGCTGAAAGTG-3′ R 5′-GTAGGGAAGGGAGCCAACAG-3′
	Tacr1 (NK1)	NM_009313	(3) F 5′-TGGACTCTGATCTCTTCCCCAACA-3′ R 5′-GGACCCAGATGACAAAGATGACCA-3′
	Tnf	NM_013693	(3) F 5′-CCCAGACCCTCACACTCAGAT-3′ R 5′-TTGTCCCTTGAAGAGAACCTG-3′
	Csf1	NM_007778 NM_001113529 NM_001113530	(3) F 5′-GGCTTGGCTTGGGATGATT-3′ R 5′-AGGCGTGGAGGGGGAAAAC-3′

(1): Integrated DNA Technologies Inc. (Iowa, USA); (2) Life Technologies Ltd. (Carlsbad, CA, USA) TaqMan probe; (3) Berger and Paige, 2005; Berger et al, 2007. GAPDH (glyceraldehyde 3-phosphate dehydrogenase); GUSB (glucuronidase, beta); TRPV1 (transient receptor potential vanilloid 1); TRPA1 (transient receptor potential ankyrin 1); VIP (vasoactive intestinal polypeptide); Adycap1 (pituitary adenylate cyclase activating polypeptide, PACAP); Sst (somatostatin); Cxcl13 (chemokine (C-X-C motif) ligand 13 or B lymphocyte chemoattractant, BLC); Il1rn (interleukin-1 receptor antagonist, IL-1RA); Tac1 (tachykinin 1); Tac3 (tachykinin 3); Tacr1 (tachykinin 1 or neurokinin-1 receptor); Tnf (tumor necrosis factor alpha); Csf1 (macrophage colony stimulating factor 1, M-CSF).

### RNA Assay

QuantiGene 2.0 Plex Assay (product name: Plex Set 21491 MOUSE 8 plex Magnetic Beads; catalog number: 321491; Affymetrix Inc./Panomics, CA, USA) was used to quantitate the mRNA levels of 1 reference gene (beta-actin) and 7 target genes (see Table 2) in total RNA isolated from distal colon sections of DSS treated and water-consuming WT and TRPA1 KO mice (for RNA isolation see *Quantitative PCR*). The assay was performed according to the manufacturer's instructions. Briefly, Luminex beads with capture probes were incubated overnight with 20 µl of the RNA sample (0.5 µg/μl) and gene specific probe set. All samples were run in duplicates. On the 2nd day, signal amplification was carried out by incubating the samples with pre-amplifiers, amplifiers and label probe. Detection was performed by adding streptavidin phycoerythrin (SAPE) to the samples and reading signal using a Magpix instrument (Luminex Corp., Austin, TX, USA). mRNA fold change normalized to the reference gene beta actin was calculated according to the manufacturer's instructions.

**Table 2:** Mouse genes detected by the Quantigene 2.0 Plex RNA Assay in distal colon homogenates of DSS-treated WT and TRPA1 KO mice.

Symbol	Name	Accession
Trpv1	transient receptor potential cation channel, subfamily V, member 1	NM_001001445
Trpa1	transient receptor potential cation channel, subfamily A, member 1	NM_177781
Actb	actin-beta	NM_007393
Adcyap1r1	adenylate cyclase activating polypeptide 1 receptor 1 (PAC1)	NM_007407
Vipr1	vasoactive intestinal peptide receptor 1 (VPAC1)	NM_011703
Vipr2	vasoactive intestinal peptide receptor 2 (VPAC2)	NM_009511
Sstr1	somatostatin receptor 1	NM_009216
Sstr4	somatostatin receptor 4	NM_009219

### Luminex Multiplex Immunoassay

The excised and frozen tissues were thawed and weighed, and immediately placed in PBS containing 10 mg/ml phenyl methyl sulfonyl fluoride (PMSF) protease inhibitor, and homogenized as described above. Then Triton X-100 was added to the samples to a final concentration of 10 mg/ml and centrifuged at 10000 g for 5 minutes to remove cell debris. Luminex Multiplex Immunoassay was performed as described previously (Nedvig et al. 2012) Briefly, the protein levels of the following cytokines/chemokines selected on the basis of a previous experiment to reveal the most important inflammatory cytokines in this model [44] were determined using customized Milliplex Map Kit (Millipore): 1. interleukin-1beta (IL-1β); 2. monocyte chemotactic protein-1 (MCP-1) also known as chemokine (C-C motif) ligand 2 (CLC2); 3. monokine induced by gamma interferon (MIG) also known as chemokine (C-X-C motif) ligand 9 (CXCL9); 4. regulated on activation, normal T cell expressed and secreted (RANTES) also known as chemokine (C-C motif) ligand 5 (CCL5). The experiment was performed according to the manufacturer's instructions. Following previous optimizations, all samples were tested undiluted in a blind-fashion. Luminex100 device was used for the immunoassay and Luminex 100 IS software for the analysis of bead median fluorescence intensity. Samples were homogenized with RPMI-1640 (GIBCO) containing 1% protease inhibitor cocktail, samples were used in 20 mg/ml concentrations. All the tests were run in duplicate. 25 µl volume of each sample, control, or standard was added to a 96-well plate (provided with the kit) containing 25 µl of antibody-coated fluorescent beads. Biotinylated secondary antibodies and streptavidin-PE were added to the plate with alternate incubation and washing steps. After the last washing step, 100 µl of the buffer was added to the wells; the plate was incubated and read on the Luminex100 array reader, using a five-PL regression curve to plot the standard curve. Data were analyzed using the MasterPlex software. Results are given in pg/g wet tissue.

### Statistics

Results are expressed as means ± SEM. For the low number of animals per group (3–5 per group), non-parametric tests were applied to evaluate the data (Kruskal-Wallis test, Mann-Whitney test). In the case of the Disease Activity Index, when comparing the two genotypes, two-way ANOVA followed by Bonferroni's post-test was used based on the number of animals (14 per genotype) and testing data distribution (normality testing by Kolmogorov-Smirnov test). The respective statistical analysis were done using GraphPad Prism 5.02 for Windows (GraphPad Software, USA). Probability values p<0.05 were accepted as significant.

## Ethical Considerations

All experimental procedures were carried out according to the 1998/XXVIII Act of the Hungarian Parliament on Animal Protection and Consideration Decree of Scientific Procedures of Animal Experiments (243/1988) and complied with the recommendations of the International Association for the Study of Pain and the Helsinki Declaration. The studies were approved by the Ethics Committee on Animal Research of University of Pécs according to the Ethical Codex of Animal Experiments and licence was given (licence numbers: BA 02/2000-2/2012 for animal experiments and BA02/2000-1/2012 for collecting human biopsy samples). All patients signed informed consent forms.

## Results

### TRPA1 and TRPV1 gene expressions in the intact and inflamed mouse and human colon

**Figure 1:**
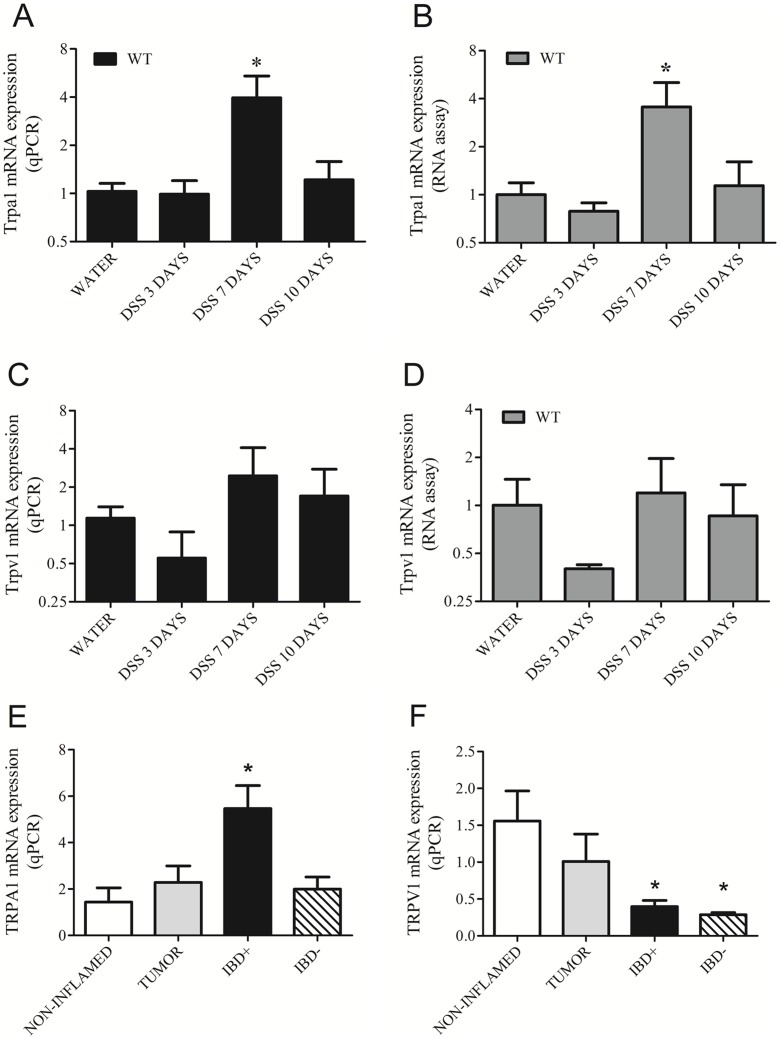
Transient Receptor Potential Ankyrin 1 (Trpa1) and Vanilloid 1 (Trpv1) gene expressions determined by (A, C) qPCR and (B, D) RNA assay in the distal colon samples of intact mice (water-drinking, non-inflamed), as well as after 3, 7 and 10 days of dextran-sulphate (DSS) administration (inflamed; n = 3-5/group). Panels **E** and **F** demonstrate TRPA1 and TRPV1 expression in human colon biopsies (non-inflamed n = 5, tumor n = 8, active inflammatory bowel diseases/IBD+ n = 5, inactive phase of colitis/IBD- n = 5). Columns represent means±SEM, *p<0.05 vs. respective non-inflamed control (Mann-Whitney U test for the murine samples, Kruskal-Wallis and Dunn's post test for the human samples).

The qPCR results reveal that Trpa1 mRNA is upregulated in mice on day 7 of the DSS treatment, compared to the water-receiving control (Figure 1A). The RNA assay results (Figure 1B) confirm the same expression pattern. Trpv1 mRNA levels in WT mice detected by qPCR (Figure 1C) and RNA assay (Figure 1D) are not affected significantly by the DSS treatment. The RNA assay detects directly mRNA as opposed to qPCR that involves an extra step of reverse transcription prone to technical variability. Matching results of these two different methods validate our data. In humans, TRPA1 gene expression is significantly upregulated in patients with active IBD, but not with inactive IBD, compared to non-inflamed samples (Figure 1E). TRPV1 mRNA significantly decreases in active IBD patients compared to the non-inflamed group. Tumor colon biopsies, used for comparison in the present study, express TRPV1 and TRPA1 local mRNA gene expression but do not exhibit significant change in their gene expression levels compared to non-inflamed controls.

### Immunohistochemical localization of TRPA1 and TRPV1 receptor proteins in the intact and inflamed mouse and human colon

**Figure 2:**
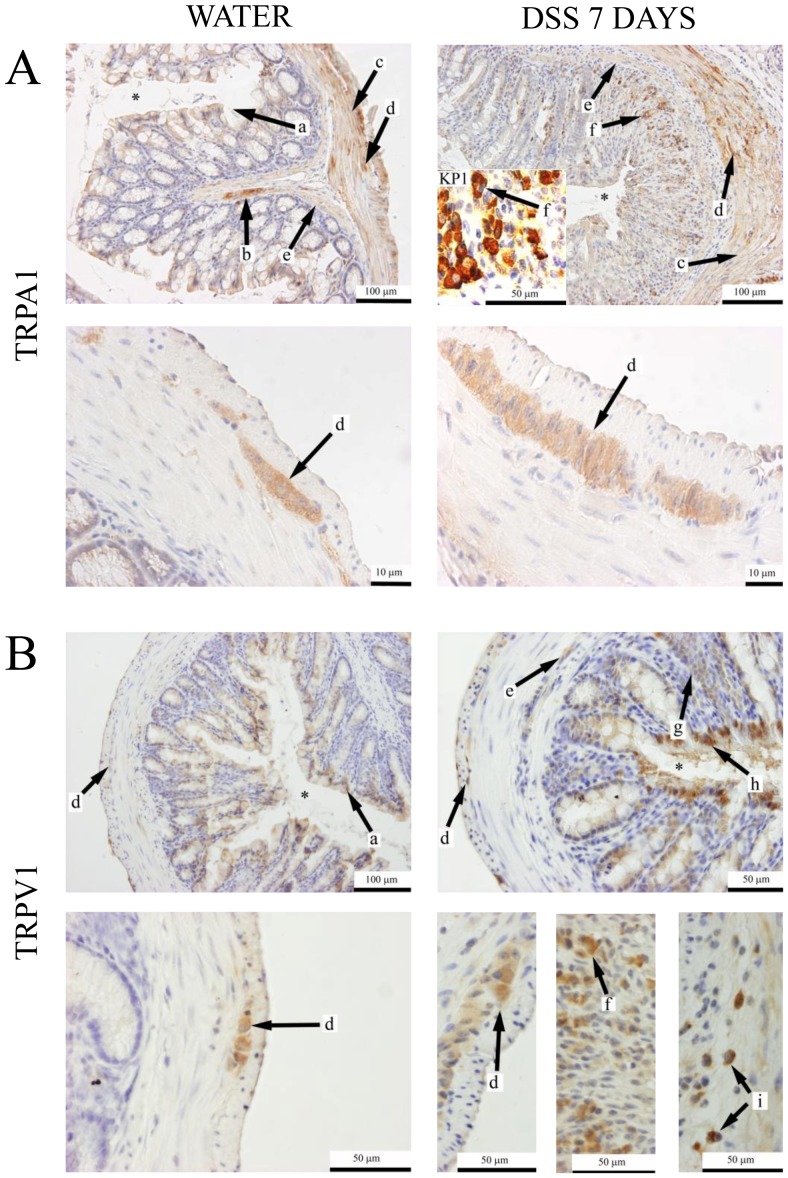
Representative immunohistochemical pictures of (A) TRPA1 and (B) TRPV1 labelling of intact, non-inflamed distal colon sections of C57Bl/6 mice drinking water or inflamed tissues of mice receiving dextran-sulphate (DSS) for 7 days. Insert: KP1 (anti-CD68) antibody-labelled macrophages. Asterix: colon lumen. Arrows: *a:* weak immunopositivity on mucosal epithelial cells; *b:* remarkable immunopositivity around mucosal nerves and blood vessels; *c:* myenteric plexus nerve fibers; *d:* myenteric plexus ganglia; *e*: submucous plexus. *g:* infiltrating immune cells; *h:* marked immunopositivity on inflammatory leukocytes. *i:* interstitial plasma cells in the submucosa.

**Figure 3:**
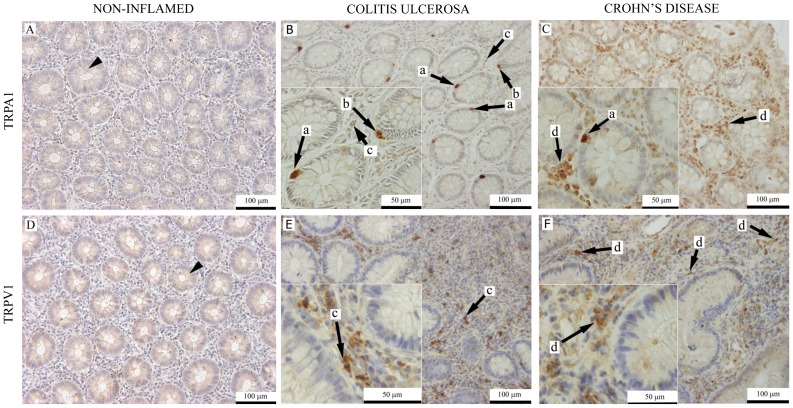
Representative photomicrographs of (A, B, C) TRPA1 and (D, E, F) TRPV1 immunohistochemically labeled sections of colon biopsies from control patients with non-inflamed colon, colitis ulcerosa and active Crohn's disease. Arrowhead: crypt epithelial cells. Arrows: *a*: granulated gland cells corresponding to neuroendocrine cells; *b*: Paneth cells. *c*: interstitial macrophages. d: infiltrating plasma cells and macrophages.

We provided immunohistochemical evidence that TRPA1 receptor immunopositivity is located on mucosal epithelial cells, around mucosal nerves and blood vessels, myenteric nerve fibers and ganglia in distal colon sections of water-treated C57BL/6 mice (Figure 2A). In addition, distal colon sections of DSS-treated mice also show TRPA1 immunostaining on interstitial macrophages and the stuctures of the submucosal plexus. The presence of infiltrating macrophages in the mucosa is confirmed by KP-1 (anti-CD68) immunostaining. TRPV1 receptor is detected on enteric ganglia, and weak immunopositivity is present on epthelial cells in distal colon sections of water-receiving animals. DSS-treated mice show TRPV1 immunopositivity also on the submucosal plexus, mucosal macrophages, submucosal plasma cells and marked immunopositivity is detected on leukocytes near the epithelial layer (Figure 2B). In distal colon biopsies of non-inflamed control patients, poor TRPA1 and TRPV1 immunopositivity was found in the crypt epithelium (Figure 3A,D). In IBD patients, TRPA1 immunopositivity is present on neuroendocrine cells of intestinal crypts, Paneth cells, macrophages and interstitial plasma cells (Figure 3B,C). TRPV1 immunopositivity is also present on interstitial plasma cells and macrophages. Sporadic, weak TRPV1 immunopositivity is detected on some neuroendocrine cells (Figure 3E,F).

### Disease Activity Index in the mouse model

**Figure 4:**
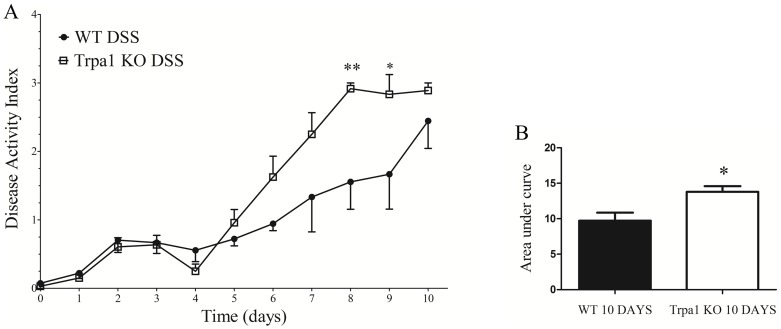
Disease Activity Index of mice calculated daily on the basis of body weight, stool consistency and fecal blood content (see Table 1) (A) shown every day and (B) as areas under curve during the total 10-day-experiment. Wildtype (WT) and TRPA1 knock-out (KO) mice were orally administered 2% dextran-sulfate (DSS) and compared to intact, water-consuming control animals. Data points represent means±SEM (n = 14-15). In panel A **p<0.01 and *p<0.05, KO DSS vs. WT DSS (two-way ANOVA with Bonferroni's post-test); effect of genotype (column factor) p<0.0001; in panel B *p<0.05 (Mann-Whitney U test).

Disease Activity Index (DAI) is significantly increased by the genetic lack of the functional TRPA1 receptor during the 10-day experiment (Fig. 4A). Disease Activity Index values in KO animals are significantly elevated on the 8th and 9th days compared to their WT counterparts. TRPA1 KO animals show a significantly greater area under the curve value compared to the WTs during the 10-day DSS-treatment (Figure 4B). In the DSS groups on the 10th day, 1 out of 4 KO animal had been lost before terminating the experiment (preterm death) on the 9th day, while all WTs survived. Taking into consideration ethical and animal welfare aspects, the authors propose DSS drinking only for a maximum of 7 days followed by 3 days of water drinking in future experiments. This should be sufficient to induce colitis since the 7-day 2% DSS colitis model has been proven to work well in our laboratory [44], [54]. However, a total duration of 10 days is necessary to show the difference between WT and TRPA1 KO mice, as demonstrated by our data.

### Semiquantitative histological analysis of the intact and inflamed mouse colon samples

**Figure 5:**
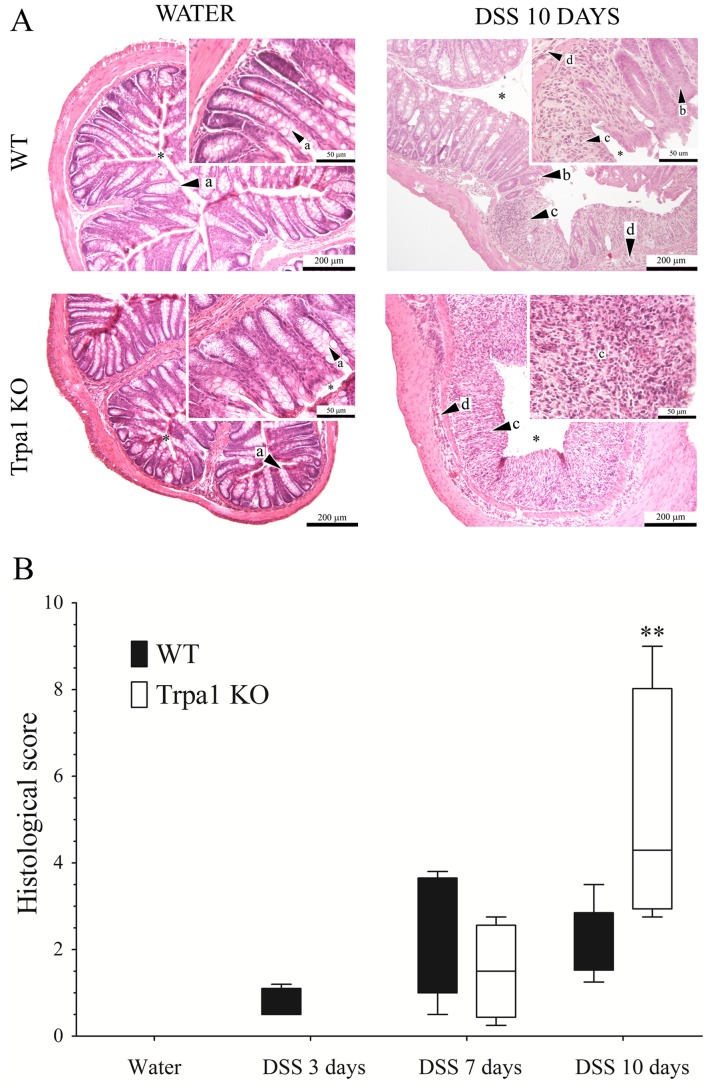
Representative light micrographs of distal colon samples of (A) water-treated non-inflamed wildtype (WT) and TRPA1-deficient (Trpa1 KO) mice, as well as after 10 days of dextran-sulphate (DSS) drinking. Sections are stained with haematoxylin-eosin. Arrows show *a)* intact crypts, *b)* damaged crypt, *c)* mucosal neutrophil infiltration, *d)* submucosal neutrophil infiltration, asterisk shows the colon lumen. Magnification: 100×; inserts: 400×. Panel (**B**) demonstrates the box plots of the semiquantitative histopathological scores of distal colon sections of water-treated non-inflamed wildtype (WT) and TRPA1-deficient (Trpa1 KO) mice, as well as after 3, 7 and 10 days of dextran-sulphate (DSS).

Compared to a histopathological picture of the non-inflamed colon structure showing intact crypts and normal mucosal epithelial layer, 20 mg/ml DSS drinking results in inflammation and tissue damage (Figure 5A). There is a significantly elevated mucosal and submucosal neutrophil infiltration, loss of crypts and disintegration of the mucosal structure in both genotypes. The severity and the extent of these characteristic histopathological alterations are significantly increased in the lack of the TRPA1 receptor on the 10th day compared to the respective KO group (histological score: Fig. 5B).

### Cytokine gene expressions in the intact and inflamed mouse colon

**Figure 6:**
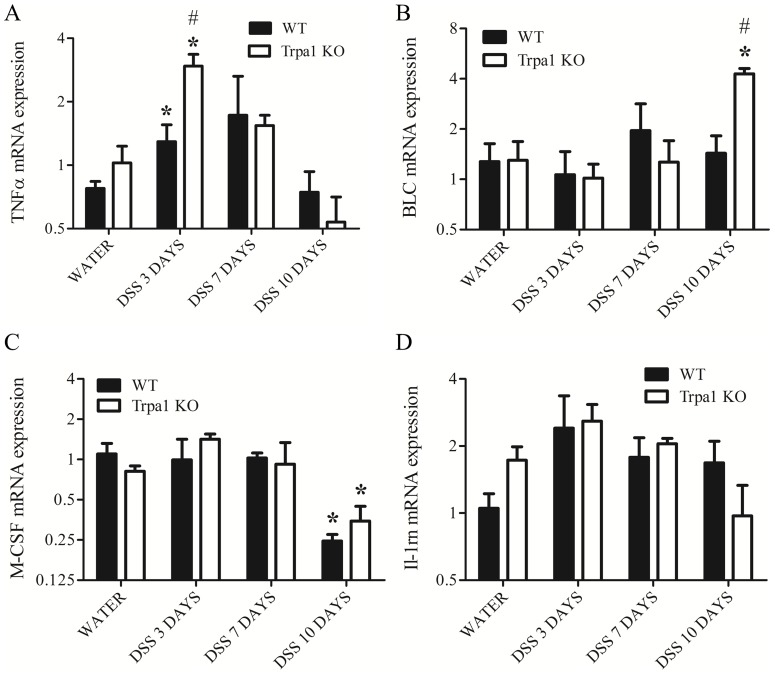
Gene expression profiles of inflammatory cytokines, such as (A) TNF-alpha, (B) BLC, (C) M-CSF, (D) IL-1rn in the distal colon homogenates of water-treated non-inflamed wildtype (WT) and TRPA1-deficient (Trpa1 KO) mice, as well as after 3, 7 and 10 days of dextran-sulphate (DSS) determined by quantitative PCR. Columns represent means±SEM. *p<0.05, **p<0.01 vs. respective water-receiving control; #p<0.05, #p<0.01 WT vs. KO (n = 3-5/group, Mann-Whitney U test).

The mRNA transcripts of the cytokines/chemokines TNFα, BLC, M-CSF and IL-1 receptor antagonist are detected in distal colon homogenates of water-receiving intact and DSS-treated colitis mice (Figure 6). TNFα gene expression is upregulated on day 3 in both genotypes compared to the non-inflamed controls, showing a greater increase in KO animals compared to their WT counterparts (Figure 6.A). BLC expression is upregulated in KO mice on the 10th day of the DSS treatment compared to both respective water-treated and WT control groups (Figure 6.B). M-CSF mRNA expression is downregulated by DSS in both genotypes on the 10th day with no difference between WTs and KOs (Figure 6.C). IL-1rn gene expression is not significantly altered, compared to respective water-consuming groups (Figure 6.D).

### Cytokine protein expressions in the intact and inflamed mouse colon

**Figure 7:**
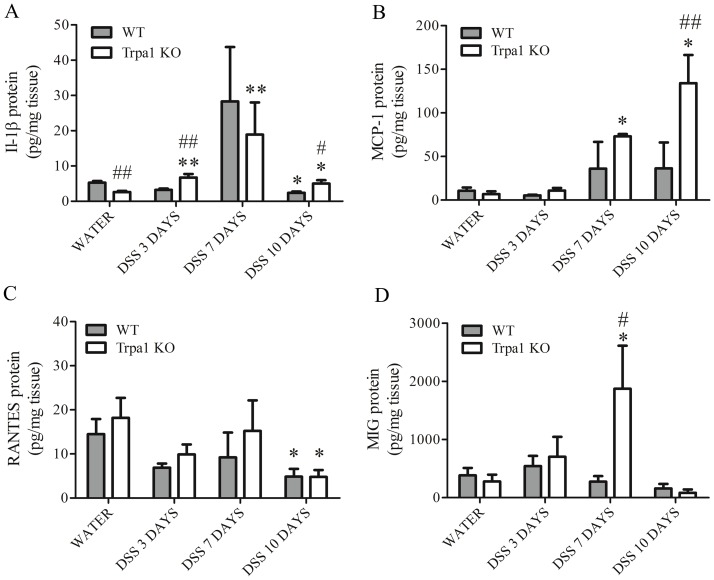
Protein expression profiles of inflammatory cytokines, such as (A) IL-1b, (B) MCP-1, (B) RANTES, and (C) MIG in the distal colon homogenates of water-treated non-inflamed wildtype (WT) and TRPA1-deficient (Trpa1 KO) mice, as well as after 3, 7 and 10 days of dextran-sulphate (DSS) determined by multiplex bead array. Columns represent means±SEM. *p<0.05, **p<0.01 vs. respective water-receiving control; #p<0.05, #p<0.01 WT vs. KO (n = 3-5/group, Mann-Whitney U test).

IL-1β protein expression detected by the Luminex multiplex bead array is significantly lower in water-treated KO animals compared to their WT counterparts. DSS treatment elevated its expression in both genotypes. IL-1β is significantly upregulated in KO animals on days 3 and 7 compared to the respective water-consuming controls. On day 10, IL-1β expression decreasesin KOs compared to the 7th day, but remains significantly higher compared to respective water-consuming controls. In WTs, its expression is downregulated on the 10th day compared to respective water-consuming controls (Figure 7.A). MCP-1 is significantly upregulated in DSS-treated KOs on days 7 and 10 compared to the respective water-receiving control and also in comparison to respective WTs on the 10th day (Figure 7.B).

RANTES expression is significantly downregulated by the DSS treatment on the 10th day in both genotypes compared to the respective water-consuming groups (Figure 7.C).

MIG expression is significantly increased in KOs on the 7th day compared to both respective water-consuming KO and DSS treated WTs (Figure 7.D).

### Gene expression of Trpv1, neuropeptides and their receptors in the intact and inflamed mouse colon

**Figure 8:**
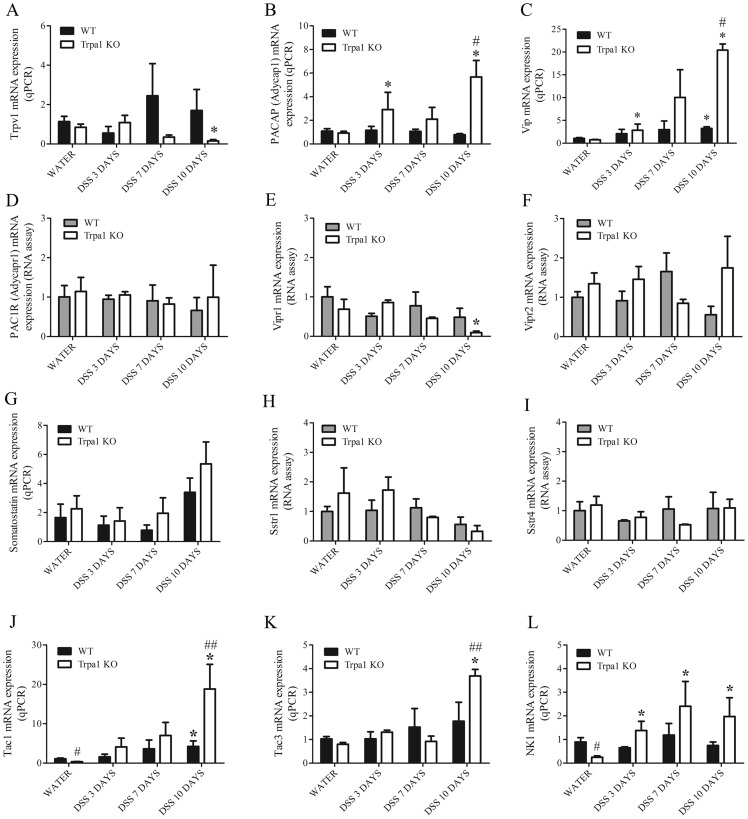
Gene expression profiles of (A) TRPV1 receptor, sensory neuropeptides, such as (B) PACAP, (C) VIP, and their receptors (D) PAC1R, (E) VIPR1, (F) VIPR2, as well as another sensory neuropeptide (G) somatostatin and its receptors (H) SSTR1, (I) SSTR4, and tachykinins (J) TAC 1, (K) TAC 3, as well as their receptor NK1. Gene expression was determined in the distal colon homogenates of water-treated non-inflamed wildtype (WT) and TRPA1-deficient (Trpa1 KO) mice, as well as after 3, 7 and 10 days of dextran-sulphate (DSS) determined by multiplex bead array. Columns represent means±SEM. *p<0.05, **p<0.01 vs. respective water-receiving control; #p<0.05, #p<0.01 WT vs. KO (n = 3-5/group, Mann-Whitney U test).

TRPV1 mRNA detected by qPCR is not significantly altered by the DSS treatment in WT mice. In KO animals, however, Trpv1 gene expression is significantly downregulated on the 10th day (Figure 8.A).

The mRNAs of the neuropeptides somatostatin, PACAP and VIP, along with their receptors SSTR1 and SSTR4 (somatostatin receptors 1 and 4, respectively), specific PACAP receptor PAC1, PACAP/VIP receptors VPAC1 and VPAC2 are expressed in both the non-inflamed and inflamed distal colon samples (Figure 8.B–I). In water-receiving intact animals, there is no difference between the neuropeptide and receptor gene expression profiles of WTs and TRPA1 KOs. Upon DSS treatment, PACAP (Adycap1) mRNA is significantly upregulated in KO, but not in WT animals compared to respective water-consuming controls (Figure 8.B). On the 10th day, PACAP (Adycap1) gene expression is significantly increased in TRPA1 KOs compared to their respective WT counterparts. VIP gene expression shows a significant increase in both genotypes during the whole DSS-treatment period compared to respective water-treated controls, but on the 10th day, it shows a 27-fold increase in KO mice (Figure 8.C).

PAC1 (Adycapr1), as well as VPAC1 (Vipr1) and VPAC2 (Vipr2) are detected in water- and DSS-treated WT and KO animals. VPAC1 is downregulated in the KO group receiving DSS for 10 days compared to the respective water-treated animals. Levels of PAC1 and VPAC2 are not affected significantly by the genotype and the DSS treatment (Figure 8.D-F).

Somatostatin gene expression does not change in either genotype by the DSS treatment, and does not present a significant difference between genotypes. Similarly, Sstr1 and Sstr4 (somatostatin receptors 1 and 4, respectively) are not significantly altered by the DSS treatment and the lack of TRPA1 (Figure 8.G). PAC1R (Adycapr1), VPAC1 (Vipr1), VPAC2 (Vipr2), Sstr1 and Sstr4 mRNA was directly detected by RNA assay lacking reverse transcription's technical variability.

The tachykinin genes Tac1 (encoding substance P and NKA), Tac3 (encoding neurokinin B) and the Tacr1 (NK1 receptor) gene are expressed in both the non-inflamed and the DSS-treated distal colon. Gene expression levels of substance P (SP) and NK1 receptors, but not neurokinin B are significantly downregulated in water-receiving control KO mice compared to their WT counterparts. Upon DSS treatment, Tac1 mRNA expression is upregulated in both genotypes. On the 10th day, Tac1 gene expression is significantly higher in KOs compared to their WT counterparts. Tac3 mRNA is upregulated in KO mice on the 10^th^ day compared to both respective water-treated controls and WTs. NK1 mRNA is significantly upregulated by the DSS treatment only in KOs on day 3, 7 and 10 (Figure 8.J-L).

## Discussion

We have found TRPA1 and TRPV1 gene expression and immunostaining in the mouse and human colon. TRPA1, but not TRPV1 mRNA was significantly upregulated in response to inflammation. Protective role of TRPA1 was clearly demonstrated in DSS colitis based on the Disease Activity Index and histological score and supported by the TRPA1-mediated downregulation of proinflammatory neuropeptides and cytokines.

The role of neuronal TRPA1 in mediating visceral pain is well established [7], [8], [49], [55]–[58]. TRPA1 antagonists have reached clinical trials for the treatment of inflammatory, neuropathic and visceral pain [59]. Contribution of TRPA1 to the pathogenesis of IBD, however, remains unclear with literature data indicating pro- and antiinflammatory effects or no influence [39], [48], [49]. Water-consuming WT, compared with TRPA1 KO mice, presented significantly increased basal levels of proinflammatory mediators (SP, NKA and NK1 mRNA, and IL-1β protein). The lack of TRPA1 could decrease the activating/sensitizing effect of microflora and metabolites in the gut. This reflects a basal activation of TRPA1 and correlates with its proposed colonic proinflammatory role through the release of substance P. [39], [40] In contrast, under serious inflammatory conditions we propose a shift in TRPA1's functions towards ameliorating inflammation.

Regarding the mechanisms of the TRPA1-mediated antiinflammatory function in the DSS-induced murine colitis, we have presented evidence for the decreased NK1 receptor expression in the wild-type animals compared to the knockouts. An upregulation of NK1 receptor mRNA was reported in a mustard oil (MO) induced colitis [41] in WT mice, but actions of MO in the lack of functional TRPA1 were not evaluated.

TRPA1 may exert protective effects by downregulating the gene expression of proinflammatory tachykinins SP, NKA and NKB implicated in IBD [39], [42], [43], [60]–[64]. The presence of TRPA1 decreases PACAP and VIP synthesis in the inflamed colon, while PACAP/VIP receptors are not significantly upregulated. There are recent reports on the proinflammatory role for VIP in DSS colitis, however, other literature data are heterogenous on its expression and function in experimental colitis and IBD [65]–[71]. Colonic VIP and PACAP (Adycap1) mRNA expression has various sources (enteric neurons and immune cells) and these peptides act on diverse PACAP/VIP receptor variants coupled to cell type dependent signaling pathways [65], [72]. Therefore it needs further investigation, whether the remarkable elevation of the VIP and PACAP peptide expression predicts their modulatory role in the inflammatory processes of the colon.

Based on our previous cytokine panel results [44] we detected 8 cytokines/chemokines to characterize colonic inflammation. The early inflammatory cytokine TNFα is mainly produced by macrophages, its dysregulation has been implicated in IBD and anti-TNF agents are currently in therapeutic use [73], [74]. There is evidence that TNFα is part of an early response in this colitis model [3], [75], [76]. A Th1/Th17 mediated acute inflammation with high levels of TNFα transforms into a predominantly Th2-driven chronic inflammatory response with lower levels of the cytokine. It should also be noted that protein levels may follow the day 10 decrease of mRNA levels with a delay. That is, TNFα proteins may be still present at higher concentrations than indicated by mRNA levels. Our results suggest that TRPA1 activation is likely to decrease TNFα synthesis, which also might be responsible for the receptor's inhibitory role. Similarly, TRPA1 stimulation diminishes leukocyte-attracting chemokines MIG and MCP-1. Upon DSS treatment, the presence of TRPA1 significantly downregulated IL-1β, a proinflammatory cytokine of innate immune cells and enteric neurons [77] acknowldeged to exacerbate IBD [78]. The expression of BLC playing a role in the generation of aberrant lymphoid tissue in IBD [79] elevated only in case of TRPA1 deficiency by the end of the experiment.

The immunolocalization and some protective functions of the TRPA1 channel in the mouse colon has been described by the results of Poole et al (2011) [80]. They found the activation of neuronal TRPA1 inhibits spontaneous neurogenic contractions and transit. TRPA1 would inhibit colon transit as a protection from the sensitizing/activating effect of inflammatory mediators. The upregulation of TRPA1 gene expression in murine and human IBD samples detected by us and others [41], [81], [82] can be related to enteric neuronal receptor synthesis. We detected both TRPA1 and TRPV1 on the myenteric and submucosal plexuses. Trpa1 mRNA upregulation on day 7 but not day 3 can indicate a delayed response on the gene expression level. Schmidt and coworkers [83] presented evidence for TRPA1 trafficking from intracellular stores to the plasma membrane in neurons upon stimulation. Since TRPA1 proteins already present in the cytoplasm can relocate to the cell membrane, the number of active ion channels can increase without the imminent increase of transcription. Lack of upregulation on day 10 can be the result of destruction of Trpa1-expressing structures. It should also be taken into consideration that mRNA downregulation can be followed by protein levels with a delay. Macrophage TRPA1 with a proposed antiinflammatory role [47] can also contribute to the elevated gene expression levels. It is to be noted that local Trpa1 mRNA is unlikely to include that of extrinsic sensory neurons, therefore it is not informative on the putative protective roles of TRPA1 channels synthetized in colon-innervating dorsal root ganglia.

No significant upregulation of colonic Trpv1 mRNA was detected in DSS treated WT mice, similarly as in mustard oil (MO) induced colitis [41]. Moreover, TRPV1 transcripts were downregulated in Trpa1 KO animals parallel to the enhanced inflammation upon DSS treatment. IBD patients showed decreased TRPV1 gene expression, while a study on UC patients reported no change in its local mRNA levels [84]. Opposite direction of TRPV1 gene expression alterations compared to TRPA1 may reflect disparate functions in the various cell types they are expressed in the inflamed colon. Extrinsic sensory TRPV1 receptors mediated pro- and antiinflammatory functions and were upregulated in IBD [11]–[18], [55], [85]–[87]. Its protein is translocated intraaxonally to the periphery [88], albeit there is a report on TRPV1 mRNA in peripheral axons [89]. We do not suggest the contribution of extrinsic sensory TRPV1 receptors to colonic mRNA levels since we previously described that TRPV1 expression in the skin is unaffected by chemical or surgical denervation of primary sensory afferents [90]. Furthermore, neuronal TRP mRNA levels have been shown to highly exceed those of non-neuronal cells [91], [92], as action potential generation is supposed to require higher receptor density compared to non-neuronal functions. Taken together, we suggest that enteric neurons largely contribute to TRP mRNA detected in our colon samples. Literature data are accumulating for the presence of intrinsic enteric TRPV1 in the gut but its excitatory/secretory functions remain to be elucidated [13], [39], [60], [93]–[99].

Inflammation evoked accumulation of TRPA1 and TRPV1 positive leukocytes. A large number of these cells in the mucosal layer were identified by KP-1 (anti-CD68) immunostaining as macrophages. Infiltrating macrophages play a role in the initiation and development of IBD [100]. There are *in vitro* data on a peculiar partially anti-inflammatory role of macrophage TRPA1/V1 channels, but their function in inflammatory processes *in vivo* has not been investigated [31], [47].

In non-inflamed control biopsies of patients, weak TRPA1 and TRPV1 immunopositivity was found in crypt epthelial cells, similarly to the mouse. In IBD biopsies, neuroendocrine cells of intestinal crypts were stained with the anti-TRPA1 antibody. Likewise in the mouse, human interstitial leukocytes showed TRPV1 and TRPA1 immunopositivity. Paneth cells and granulated neuroendocrine cells in crypts of Lieberkühn presented remarkable TRPA1 immunopositivity in human distal colon sections of IBD patients. It can be hypothesized that colonic neurendocrine TRPA1 receptors may modulate colitis through serotonin and melatonin release [101]–[103].

Localization of the TRPA1 channel on different cell types of the colon (extrinsic sensory neurons, enteric neurons, neuroendocrine and immune cells), its upregulation in inflammation similarly in humans and mice, as well as its clearly ameliorating role in the chronic colitis mouse model suggests important protective functions in IBD. The mechanisms underlying the anti-inflammatory actions of TRPA1 in colitis can be explained by decreasing proinflammatory SP, NKA, NKB and NK1 receptor expression, as well as inhibiting the synthesis of inflammatory cytokines and chemokines presumably derived from macrophages. Corresponding TRPA1 expression patterns in murine and human colon samples suggest an important translational relevance of our functional experimental findings. The proposed protective roles of TRPA1 indicate future prospects in inflammatory bowel diseases research.

## Supporting Information

Figure S1 Photomicrographs of (A) mouse and (B) human colon sections immunohistochemically labeled by TRPA1 and TRPV1 antibody preadsorbed with (second column) or without (first colmn) the respective blocking (immunizing) peptide. Magnification: 200x.
(TIF)

Table S1 Disease activity index scoring chart.
(DOCX)

Table S2 Histopathological semiquantitative scoring chart (severity of inflammation and extent of inflammation does not have grade 4 for the purpose of simplifying the ranges of categories)
(DOCX)

Table S3 The ARRIVE (Animal Research: Reporting In Vivo Experiments) Guidelines Checklist completed for the current manuscript.
(PDF)
