# Supplementary figures and images for: Upregulation of the Transient Receptor Potential Ankyrin 1 Ion Channel in the Inflamed Human and Mouse Colon and Its Protective Roles
Source: PLoS One. 2014 Sep 29;9(9):e108164. doi: 10.1371/journal.pone.0108164 (PMC4180273; doi:10.1371/journal.pone.0108164)

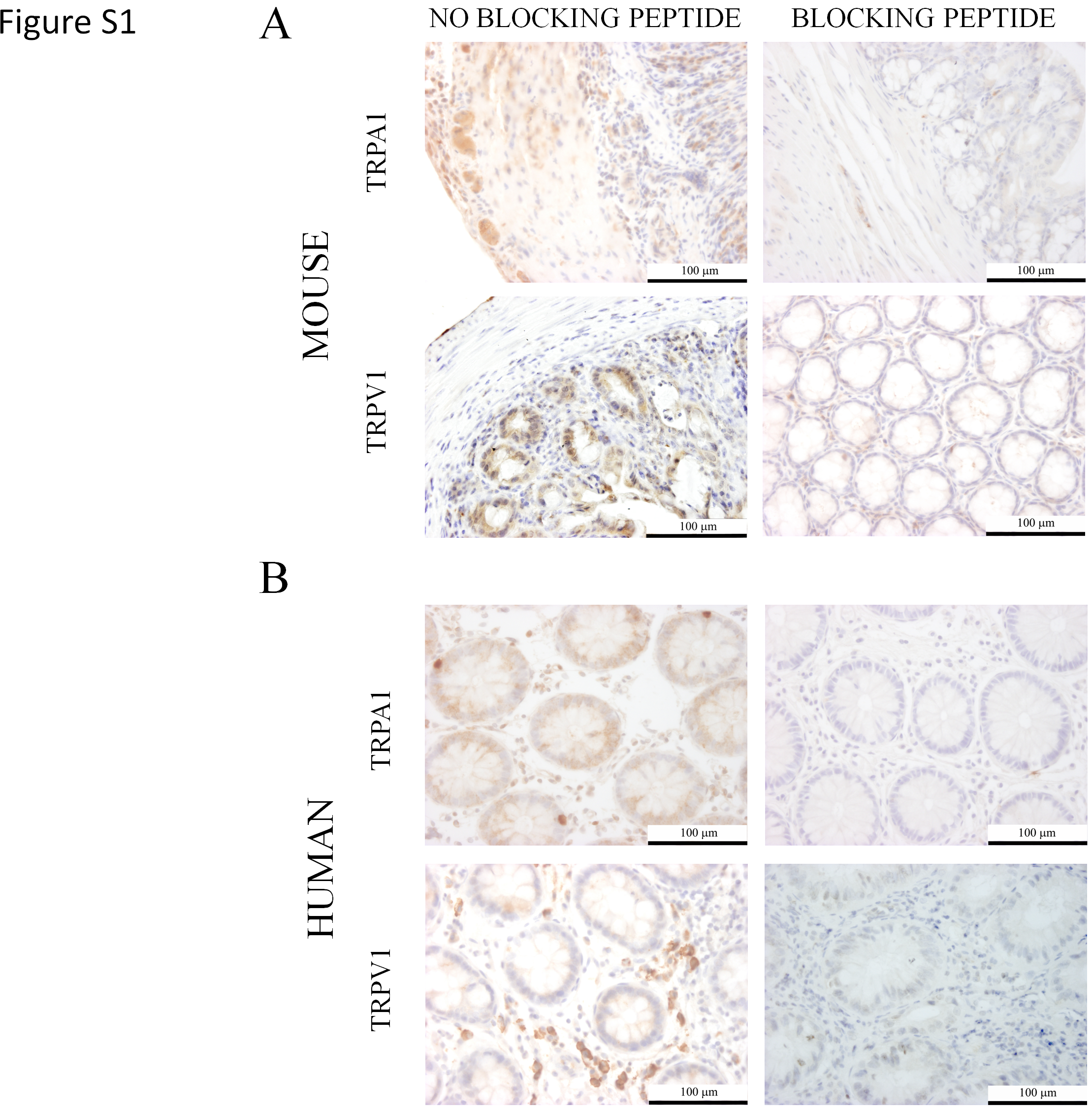

Supplement: Figure S1 — Photomicrographs of (A) mouse and (B) human colon sections immunohistochemically labeled by TRPA1 and TRPV1 antibody preadsorbed with (second column) or without (first colmn) the respective blocking (immunizing) peptide. Magnification: 200x. (TIF) [file pone.0108164.s001.tif]
