# Supplementary material for: Upregulation of the Transient Receptor Potential Ankyrin 1 Ion Channel in the Inflamed Human and Mouse Colon and Its Protective Roles
Source: PLoS One. 2014 Sep 29;9(9):e108164. doi: 10.1371/journal.pone.0108164 (PMC4180273; doi:10.1371/journal.pone.0108164)
Supplement: Table S1 — Disease activity index scoring chart. (DOCX) [file pone.0108164.s002.docx]

**Table S1.**

| **Score** | **0** | **1** | **2** | **3** | **4** |
| --- | --- | --- | --- | --- | --- |
| **Body weight loss** | 0-0.9% | 1-5% | 5.1-10% | 10.1-20% | >20.1% |
| **Fecal blood content (Hemocare test)** | negative | light blue stains | blue stain | bloody patches/blue stain | gross bleeding |
| **Stool consistency** | normal | normal/soft | soft | soft/watery | watery |
