# Supplementary material for: Upregulation of the Transient Receptor Potential Ankyrin 1 Ion Channel in the Inflamed Human and Mouse Colon and Its Protective Roles
Source: PLoS One. 2014 Sep 29;9(9):e108164. doi: 10.1371/journal.pone.0108164 (PMC4180273; doi:10.1371/journal.pone.0108164)
Supplement: Table S2 — Histopathological semiquantitative scoring chart (severity of inflammation and extent of inflammation does not have grade 4 for the purpose of simplifying the ranges of categories) (DOCX) [file pone.0108164.s003.docx]

**Table S2.**

| **Score** | **0** | **1** | **2** | **3** | **4** |
| --- | --- | --- | --- | --- | --- |
| **Severity of inflammation** | normal | mild | moderate | severe | - |
| **Damage of crypts** | none | one third of basal | two thirds of basal | crypts disappeared | mucosa disappeared |
| **Extent of inflammation** | not inflamed | mucosa only | submucosa | whole gut wall | - |
| **Percent of damaged area** | 0% | 1-25% | 26-50% | 51-75% | 76-100% |
